# Kamishoyosan and Shakuyakukanzoto promote recovery from paclitaxel-induced neurite retraction in PC12 cells

**DOI:** 10.1186/s40780-017-0090-y

**Published:** 2017-07-21

**Authors:** Ken Konaka, Kota Moriyama, Takumi Sakurada, Naoto Okada, Masaki Imanishi, Yoshito Zamami, Kazuyoshi Kawazoe, Shuji Fushitani, Keisuke Ishizawa

**Affiliations:** 1 Department of Pharmacy, Tokushima Municipal Hospital, 2-34 Kitajyosanjima, Tokushima, 770-0812 Japan; 2 Department of Pharmacy, Shimane University Hospital, 89-1 Enya, Izumo, 693-0021 Japan; 3 Department of Pharmacy, Tokushima University Hospital, 2-50-1 Kuramoto, Tokushima, 770-8503 Japan; 4 Department of Clinical Pharmacology and Therapeutics, Institute of Biomedical Sciences, Tokushima University Graduate School, 2-50-1 Kuramoto, Tokushima, 770-8503 Japan; 5 Department of Clinical Pharmacy Practice Pedagogy Institute of Biomedical Sciences, Tokushima University Graduate School, 1-78-1 Shomachi, Tokushima, 770-8505 Japan

**Keywords:** Paclitaxel, Neuropathy, Kamishoyosan, Shakuyakukanzoto, PC12 cells

## Abstract

**Background:**

In chemotherapy, the full round of treatment must be completed as scheduled to achieve the strongest therapeutic effect. However, peripheral neuropathy, a severe side effect of the chemotherapeutic agent paclitaxel, can force the premature discontinuation of treatment. As some kampo practitioners have suggested that it may be possible to counteract such side effects, we analyzed the effects of Kamishoyosan, Shakuyakukanzoto, and Goshajinkigan in an in vitro model of paclitaxel-induced peripheral neuropathy.

**Methods:**

Paclitaxel-treated PC12 cells were assessed for neurite length and performed Western blot analysis for growth-associated protein-43 (GAP-43) and light neurofilament protein (NF-L) levels in the presence of nerve growth factor (NGF); they were re-assessed, with additional testing for acetylcholinesterase levels, after application of one of the kampo. We also compared phosphorylation of extracellular signal-regulated kinase (Erk)1/2 and Akt via Western blot analysis. About effect of kampo to anticancer efficacy, we confirmed cell cytotoxicity in A549 cells using MTT assay.

**Results:**

Addition of Kamishoyosan or Shakuyakukanzoto, but not Goshajinkigan, significantly improved neurite length and GAP-43 and NF-L levels from paclitaxel-treated PC12 cells, relative to those of only NGF-treated PC12 cells. The promoting effect of Kamishoyosan and Shakuyakukanzoto in neurite outgrowth is confirmed when NGF promoted neurite outgrowth, and it was inhibited partially when Erk1/2 and Akt were blocked by Erk1/2 inhibitor or Akt inhibitor alone. Furthermore, neurite outgrowth induced by TJ24 and TJ68 was inhibited more strongly when Erk1/2 inhibitor and Akt inhibitor were treated at the same time. NGF with Kamishoyosan or Shakuyakukanzoto promoted the proportion of phosphorylated Erk1/2 and phosphorylated Akt compare with NGF only. On the other hand, Kamishoyosan or Shakuyakukanzoto didn’t influence cytotoxicity of paclitaxel in A549 cells.

**Conclusions:**

Kamishoyosan or Shakuyakukanzoto promotes neurite outgrowth with NGF via increasing the proportion of phosphorylated Erk1/2 and phosphorylated Akt in PC12 cells. The effect applies to recovery from paclitaxel-induced axonal involvement and might promote recovery from paclitaxel-induced neuropathy without influence of anticancer effect of paclitaxel.

## Background

In chemotherapy, dose intensity is highly important, since antitumor agents are ineffective, but still carry significant risk, when the amount delivered is suboptimal [1]. Paclitaxel, a key chemotherapeutic used against many solid cancers, induces neuropathy as a side effect as frequently as 50% of the time in a cumulative, dose-dependent manner [2, 3]. This neurotoxicity significantly and negatively influences treatment, and may necessitate shortening or otherwise limiting such treatment. Paclitaxel promotes microtubule assembly, which damages both cancer cells and neurons alike. When damaging neurons, it does not normally penetrate the blood brain barrier, but instead produces peripheral neuropathy [4]. Shakuyakukanzoto, an herbal formula utilized in kampo medicine, is currently approved for the treatment of muscle spasms. Recently, Hidaka et al. reported that Shakuyakukanzoto may suppress paclitaxel-induced allodynia in vivo, though they were unable to elucidate the mechanisms underlying this effect [5]. Another formula, Goshajinkigan, has also been reported to potentially reduce oxaliplatin-induced peripheral neuropathy [6, 7]. The present study aimed to test whether Shakuyakukanzoto, Kamishoyosan, which which also contains all the ingredients of Shakuyakukanzoto, or Goshajinkigan could promote recovery from paclitaxel-induced neuropathy. We further aimed to elucidate the mechanisms underlying these potential effects.

## Methods

### Materials

PC12 cells, penicillin, and streptomycin were purchased from DS Pharma Biomedical Co., Ltd. (Osaka, Japan). Dulbecco’s modified Eagle medium (DMEM) and the Amplite colorimetric acetylcholinesterase assay kit were purchased from NACALAI TESQUE, INC (Kyoto, Japan). Fetal bovine serum (FBS) and horse serum (HS) were purchased from Biowest (Nuaillé, France). Paclitaxel, NGF, and GAP-43 antibodies were purchased from Merck Ltd. (Tokyo, Japan). Kamishoyosan (TJ24), Shakuyakukanzoto (TJ68), and Goshajinkigan (TJ107) were purchased, along with their 3D–HPLC charts, were provided by TSUMURA & CO. (Tokyo, Japan). We purchased MTT (3-(4,5-di-methylthiazol-2-yl)-2,5-diphenyltetrazolium bromide, yellow tetrazole) from Wako (Osaka, Japan) and anti-neurofilament light protein (NF-L) antibodies from Nova Biomedical (Waltham, USA). We purchased antibodies targeting phospho-Akt (Ser473), Akt, Phospho-p44/42 extracellular signal-regulated kinase (Erk)1/2 (Thr202/Tyr204), Erk1/2, and β-actin, as well as 2-(4-Morpholinyl)-8-phenyl-1(4H)-benzopyran-4-one hydrochloride (LY294002) and 1,4-diamino-2,3-dicyano-1,4-bis[2-aminophenylthio] butadiene (U0126) from Cell Signaling Technology Japan (Tokyo, Japan).

### Preparation of TJ24, TJ68, and TJ107 (kampo medicines)

We added 2.5 g of each granule-based formulation to 100 mL boiling water and allowed the samples to dissolve as much as possible (Table 1). After filtering the insoluble material, we centrifuged the mixtures at 10,000×*g* for 20 min at 25 °C and collected their supernatants. These were diluted with culture medium to 1 mg granule-based formulation/mL (per manufacturer’s recommendation) for this experiment.

**Table 1:** Components of the kampo medicines TJ24, TJ68, and TJ107

Extractive component of kampo medicine		
TJ24	①Bupleurum Root (1.5 g) ②Paony Root (1.5 g) ③Atractylodes Lancea Rhizoma (1.5 g) ④Poria Sclerotium (1.5 g) ⑤Japanese Angelica root (1.5 g) ⑥Moutan Bark (1 g) ⑦Gardenia Fruit (1 g) ⑧Glycyrrhiza (0.75 g) ⑨Ginger (0.5 g) ⑩Japanese Mint (0.5 g)	TJ24 (7.5 g) contains dryness extract (2.0 g) of mix herbal medicine for the percentage following.
TJ68	①Paony Root (1.5 g) ②Glycyrrhiza (1 g)	TJ68 (7.5 g) contains dryness extract (1.25 g) of mix herbal medicine for the percentage following.
TJ107	①Rehmannia Root (5 g) ②Alisma Rhizome (3 g) ③Achyranthes Root (3 g) ④Poria Sclerotium (3 g) ⑤Cornus Fruit (3 g) ⑥Moutan Bark (3 g) ⑦Dioscorea Rhizome (3 g) ⑧Cinnamon Bark (1 g) ⑨Plantago Seed (3 g) ⑩Aconitie Root (1 g)	TJ68 (7.5 g) contains dryness extract (4.5 g) of mix herbal medicine for the percentage following.

Kampo medicines were diluted with culture medium to 1 mg/mL, per manufacturer’s recommendations

### Cell culture

Rat adrenal pheochromocytoma cells (PC12 cells) were maintained on collagen I-coated dishes or plates, and cultured at 37 °C in a humidified 5% CO_2_/air atmosphere. Cells were cultured in culture medium containing DMEM plus 10% FBS, 5% HS, and 1% PC/SM. Cells were differentiated in DMEM containing 0.5% FBS, 1% HS, 1% PC/SM, and 50 ng/mL NGF (differentiation medium) for 72 h; after which they were plated at a density of 1.0 × 10^4^ cells/cm^2^ and incubated for 24 h. A549 cells, derived from lung tumor tissue, were plated at a density of 1.5 × 10^4^ cells/cm^2^ in DMEM containing 10% FBS, and 2% PC/SM. A549 cells were incubated for 3 days after seeding, then used for experimentation. To model neuropathy in the cell lines, paclitaxel (paclitaxel; 0–100 μM) diluted in culture medium was added to the medium of differentiated PC12 cells for 24 h.

### MTT assay

For the MTT assay, differentiated cells were incubated with 0.1–100 μM paclitaxel for 24 h in PC12 cells. A549 cells were incubated with 100 μM paclitaxel and either TJ24 or TJ68 for 24 h. After washing cells once with DMEM, they were incubated in 0.5 mg/mL MTT for 4 h, and then lysed in 500 μL DMSO. For each well, 200-μL supernatant was transferred to a 96-well plate, and absorbance at 570 nm measured using a microplate reader (TECAN InfiniteF50R) [8].

### Evaluation of neurite growth

Cells were captured using phase contrast microscopy (NIKON ECLIPSE TS100) and Digital Sight (NIKON DS-Fi2). Images were imported to ImageJ (NIH, Baltimore, Maryland, USA) to measure neurite length, which was defined as the total length of the neurite cylinder axis. The ratio of neurites to number of cells in the picture was also measured. Images were captured following the incubation of PC12 cells in differentiation and culture medium for 24 h; following the incubation of differentiated cells with 0.1–100 μM paclitaxel for 24 h in PC12 cells; and following the incubation of differentiated cells exhibiting paclitaxel-induced neurite retraction (1 μM) in differentiation medium with TJ24, TJ68, and NGF for 72 h (without paclitaxel).

### Western blot analysis

PC12 cells were rinsed with DMEM and lysed in ice-cold lysis buffer consisting of 20 mmol/L Tris–HCl (pH 7.5), 150 mmol/L NaCl, 1 mmol/L Na_2_EDTA, 1 mmol/L EGTA, 1% Triton X-100, 2.5 mmol/L sodium pyrophosphate, 1 mmol/L β-glycerophosphate, 1 mmol/L sodium orthovanadate, 1 μg/mL leupeptin, and 1% phenylmethylsulfonyl fluoride; lysed cells were then homogenized. After boiling for 5 min, equal amounts (20 μL) of the protein samples were subjected to sodium dodecyl sulfate-polyacrylamide gel electrophoresis using 10% gels, and the proteins were then transferred onto nitrocellulose membranes at room temperature. The blots were probed using their respective antibodies (at 4 °C overnight), followed by the corresponding secondary antibody (room temperature, 1 h). Phosphorylated ERK1/2, ERK1/2, Phosphorylated Akt, and Akt antibody were diluted in 1% BSA/TBS-T (1:1000). Secondary antibodies were diluted in 1% skim milk/TBS-T (1:2000). GAP-43 and β-actin antibody were diluted in 1% skim milk/PBS-T (1:500, 1:1000), while secondary antibodies were diluted in 1% skim milk/PBS-T (1:1000). NF-L antibody and its secondary antibody were diluted in 1% skim milk/PBS-T (1:1000). Blots were developed using ECL Western Blotting Detection Reagent (GE Healthcare Japan) and quantified using ImageJ. These experiments were conducted at the time of neurite length measurement.

### Amplite colorimetric acetylcholinesterase assay

The Amplite kit quantifies thiocholine produced from cholinesterase-mediated acetylthiocholine hydrolysis using 5,5′-dithiobis(2-nitrobenzoic acid) (DTNB). To ascertain the function of differentiated cells, we verified the ability of TJ24, TJ68, and NGF (without paclitaxel) to recover the release of acetylcholine from cells with paclitaxel-induced neurite retraction. The manufacturer’s recommended protocol was followed; briefly, 50 μL of acetylthiocholine reaction mixture (assay buffer, DTNB, acetylthiocholine) was added to each cell lysate sample. The reaction was incubated for 20 min at room temperature and protected from light [9]. The absorbance at 410 ± 5 nm was measured with the microplate reader (TECAN InfiniteF50R).

### Statistical analysis

Results are expressed as mean ± S.E. Differences between groups were analyzed using Dunnett’s *t*-test. In all cases, *p* < 0.05 was considered statistically significant.

## Results

### Paclitaxel-induced neuropathy

**Fig. 1:**
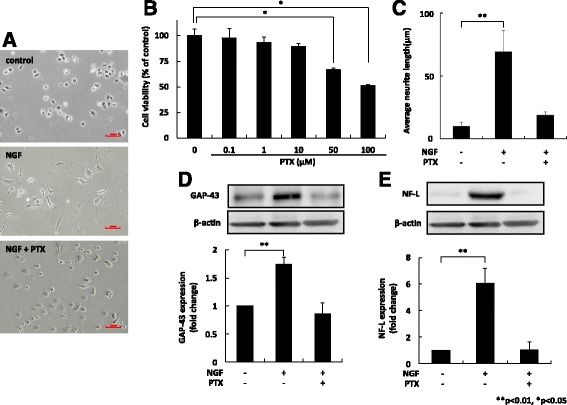
Paclitaxel (1 μM) induced neurite retraction without cytotoxicity in PC12 cells. We evaluated cell viability and neurite length after applying varying concentrations of paclitaxel to PC12 cells. A total of 1 μM paclitaxel preserved the PC12 cells based on MTT assay, but still caused neurite regression compared with NGF application only (**a**, **b**, **c**). Protein expression of the neuronal differentiation markers GAP-43 and NF-L, decreased significantly after addition of 1 μM paclitaxel (**d**, **e**). Results (mean ± S.E.) are representative of three independent experiments (*n* = 3). **p < 0.01, *p < 0.05. NGF: nerve growth factor; GAP-43: growth-associated protein 43; NF-L: light neurofilament; SE: standard error

Varying concentrations of paclitaxel were added to PC12 cells, following which cell viability and neurite length were evaluated. One (1) μM paclitaxel preserved the PC12 cells based on the MTT assay, though still produced regression of neurites equal to that observed following withdrawal of NGF (Fig. 1a–c). Protein expression of the neuronal differentiation markers GAP-43 and NF-L decreased significantly after application of 1 μM paclitaxel (Fig. 1d, e). Thus, we used a 1-μM paclitaxel dose for all subsequent experiments to represent paclitaxel-induced neuropathy.

### TJ24 and TJ68 promoted NGF-induced neurite outgrowth after paclitaxel-induced neuropathy

**Fig. 2:**
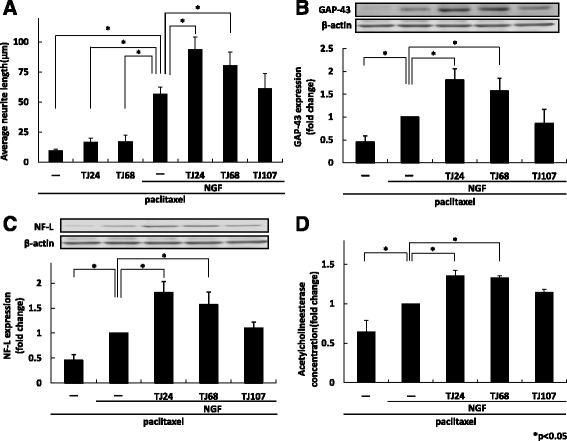
TJ24 and TJ68 with NGF improved paclitaxel-induced neuropathy morphologically and functionally. Recovery of neurites from paclitaxel-induced regression after 72 h exposure to TJ24, TJ68, NGF plus TJ24, TJ68, or TJ107 was assessed. In terms of neurite length (**a**), expression of GAP-43 (**b**), NF-L (**c**), and acetylcholine esterase concentration (**d**), treatment with NGF and TJ24 or TJ68 promoted neurite outgrowth significantly compared to NGF alone. Results (mean ± S.E.) are representative of three independent experiments (*n* = 3). * *p* < 0.05. NGF: nerve growth factor; GAP-43: growth-associated protein 43; NF-L: light neurofilament; SE: standard error

We compared neurite recovery for each formula after 72 h of exposure to paclitaxel-induced neuropathy. In terms of neurite length, treatment with NGF and TJ24 or TJ68 promoted significantly greater neurite outgrowth than NGF alone (Fig. 2a). A statistically significant increase in the expression of both GAP-43 and NF-L was also observed for the combinations of NGF and TJ24 or TJ68 (Fig. 2b, c). Moreover, both combinations increased acetylcholinesterase activity significantly relative to NGF alone (Fig. 2d). TJ107, however, failed to significantly affect any of the tested parameters. These data indicate that TJ24 or TJ68 in combination with NGF enhanced recovery after paclitaxel-induced neurite regression.

### TJ24 and TJ68 alter NGF-induced phosphorylation

**Fig. 3:**
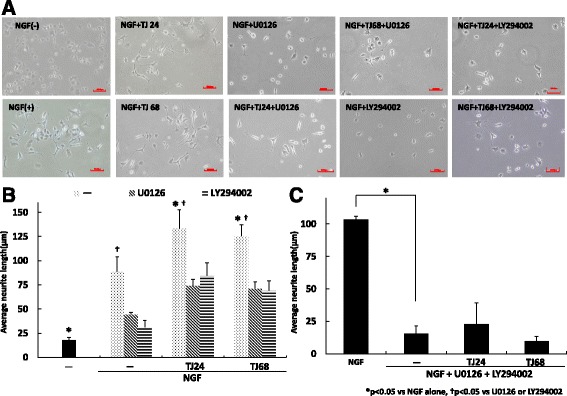
NGF with TJ24 or TJ68 alters both the ERK1/2 and Akt pathways. We analyzed neurite outgrowth after treatment with NGF alone, NGF and TJ24, or NGF and TJ68. Neurite outgrowth was also examined when the Erk1/2 inhibitor U0126, Akt inhibitor LY294002, or both were added to each previous condition for 72 h. Incubation with TJ24 or TJ68 promoted neurite outgrowth significantly compared to NGF alone (**a**, **b**). 0126 or LY294002 alone inhibited the promotion of neurite outgrowth by TJ24 or TJ68 partially. When both inhibitors were administered together, neurite outgrowth was significantly suppressed even following the addition of TJ24 or TJ68 (**c**). Results (mean ± S.E.) are representative of three independent experiments (*n* = 3). *p < 0.05 vs NGF alone, ^†^ *p* < 0.05 vs U0126 or LY294002, NGF: nerve growth factor; Erk1/2: extracellular signal-regulated kinase 1/2; SE: standard error

To begin revealing the mechanism underlying the effects of TJ24 and TJ68, we measured neurite outgrowth after incubation with NGF alone, and with TJ24 or TJ68, for 72 h; we then compared these data to those collected in the presence of U0126 or LY294002, ERK1/2, and Akt inhibitors, respectively or both. Incubation with TJ24 or TJ68 promoted significantly greater neurite outgrowth than NGF alone (Fig. 3a, b). Both U0126 and LY294002 inhibited the promotion of neurite outgrowth induced by TJ24 or TJ68 (Fig. 3a, b). Further, simultaneous treatment with U0126 and LY294002 inhibited neurite outgrowth more strongly than treatment with a single agent (Fig. 3c). These results suggest that Erk/Akt signaling activity is affected by these kampo formulas.

### TJ24 and TJ68 promoted neurite outgrowth through NGF-induced phosphorylation of ERK1/2 and Akt

**Fig. 4:**
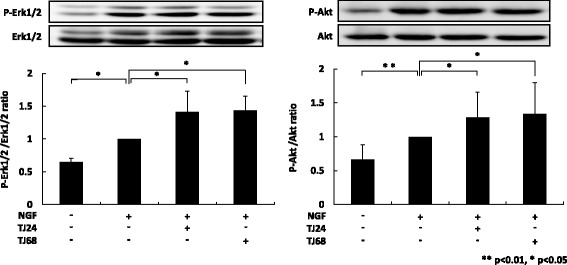
TJ24 and TJ68 promoted NGF-induced phosphorylation of Erk1/2 and Akt. We compared the proportion of phosphorylated Erk1/2 and Akt to their respective non-phosphorylated forms. Both TJ24 and TJ68 significantly increased phosphorylation of Erk1/2 and Akt compared with NGF alone. Results (mean ± S.E.) are representative of three independent experiments (*n* = 3). **p < 0.01, *p < 0.05. NGF: nerve growth factor; Erk 1/2: extracellular signal-related kinase 1/2; SE: standard error

We also investigated whether TJ24 and TJ68 increased phosphorylation of ERK1/2 and/or Akt in the process of promoting neurite outgrowth by comparing the proportion of phosphorylated ERK1/2 and Akt to their respective non-phosphorylated forms. TJ24 and TJ68 both significantly enhanced NGF-mediated phosphorylation of ERK1/2 and Akt (Fig. 4), indicating that both ERK1/2 and Akt signaling were altered.

### TJ24 and TJ68 did not affect paclitaxel-induced anticancer efficacy

**Fig. 5:**
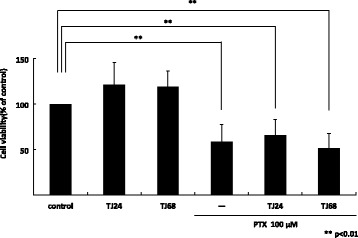
TJ24 and TJ68 did not affect paclitaxel-induced cytotoxicity in lung tumor-derived cells (A549). Cell viability after incubation for 24 h with TJ24 or TJ68 in the presence or absence of 100 μM paclitaxel was measured. Neither TJ24 nor TJ68 alone influenced cell viability, and reduced paclitaxel induced-antitumor efficacy in A549 cells compared to control. Results (mean ± S.E.) are representative of three independent experiments (*n* = 3). ** *p* < 0.01. SE: standard error

We sought to determine whether the kampo formulas also reduced the antitumor effectiveness of paclitaxel by performing a cytotoxicity assay in A549 (lung tumor-derived) cells. We compared cell viability after incubation for 24 h with TJ24 or TJ68 in the presence or absence of 100 μM paclitaxel [10]. Neither TJ24 nor TJ68 reduced the cytotoxic efficacy of paclitaxel in A549 cells (Fig. 5).

## Discussion

Though research has revealed that paclitaxel-induced neuropathy worsens with increasing frequency of administration, no adjunct therapies or altered paclitaxel administration schedules have emerged to combat the associated neuropathic damage. In clinical environments, systemic pregabalin or mecobalamin therapy is used to reduce neuropathic pain, but the effectiveness of such regimens remains questionable [11, 12]. Furthermore, pregabalin has its own potentially significant side effect—kidney dysfunction—which can also force the discontinuation of chemotherapy. Recent reports have suggested that TJ68 was effective against paclitaxel-induced neuropathy, and that TJ107 was effective against oxaliplatin-induced neuropathy, respectively, though these reports have provided limited evidence and little or no mechanistic information [5, 7]. Therefore, we felt it necessary to study these effects further utilizing a model of paclitaxel-induced neurite retraction due to its relative ease of use as well as its capacity for studying the potential mechanisms involved.

We first determined an optimal dose of paclitaxel that did not affect cell viability but induced significant neurite retraction, following which we observed that TJ24 and TJ68 in combination with NGF significantly recovered neurite outgrowth. We also noted that both morphological and functional markers were improved. However, the TJ107/NGF combination was ineffective. Our results differ from those of a previous study that analyzed oxaliplatin-induced neuropathy, though it is important to note that paclitaxel and oxaliplatin are very different drugs and induce neuropathy differently [13]. Oxaliplatin impairs the nerve cells themselves, whereas paclitaxel impairs the neuraxon [14].

**Fig. 6:**
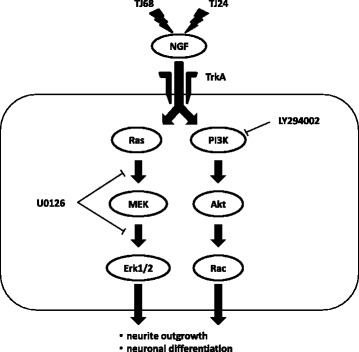
The proposed pathway via which TJ24 and TJ68 promote phosphorylation and support NGF-induced neurite outgrowth. TJ24 and TJ68 promoted neurite outgrowth by activating phosphorylation of both the Erk1/2 and Akt pathways, which in turn supports the function of NGF in extending neurites in cases of paclitaxel-induced neuropathy. NGF: nerve growth factor; Erk 1/2: extracellular signal-related kinase 1/2

Based on the need to combine NGF with TJ24 or TJ68, and the previous finding that Yokukansan extended neurite outgrowth via ERK1/2 and Akt phosphorylation [15], we focused our attention on this pathway. Although NGF-induced neurite outgrowth in PC12 cells is associated with activity of the p75NTR and TrkA pathways, this process is primarily controlled by the ERK1/2 and Akt pathways [16]. We revealed that TJ24 or TJ68 promoted neurite outgrowth induced by NGF, but it was inhibited partially when ERK1/2 and Akt were blocked by U0126 and LY294002, respectively. In addition, we revealed that neurite outgrowth induced by TJ24 and TJ68 was inhibited more strongly when U0126 and LY294002 were treated at the same time. These results also indicated that Erk and Akt work cooperatively to promote neurite outgrowth induced by TJ24 and TJ68. Thus, it seems likely that the kampo medicines promoted neurite outgrowth via activation of both the ERK1/2 and Akt pathways [17]. Indeed, we found further evidence that phosphorylation of both ERK1/2 and Akt is increased, in correlation with our other findings (Fig. 6). However, we have some limitations in this study. We used PC12 cells as neuron model, but it was different from a peripheral nerve cell a little strictly. Although PC12 cells are often used as an in vitro model of neurons in the peripheral nervous system, further investigation in dorsal root ganglion cells is required to determine the effects of TJ24 and TJ68 in vivo [18, 19]. When taking orally, we need to consider influence by metabolism in the body, but the consideration can’t be performed in this experiment. The drug disposition of kampo and specification of a single active ingredient remain unclear, and our results are limited to in vitro analysis. Further study is required in order to conclusively identify the active ingredient and clarify the efficacy of kampo medicines in vivo, as only peony root and licorice (Glycyrrhiza sp.) are common to TJ24 and TJ68. Of these, Yokukansan does not contain peony; thus, we hypothesize that licorice is the ingredient that requires further study. Because the clinical use of TJ24 or TJ68 would be concurrent with paclitaxel chemotherapy, it is also necessary to determine if either formula would interfere with its therapeutic effectiveness.

Though further work must be done to confirm our data, we noted that neither formula interfered with the ability of paclitaxel to kill A549 (tumor cell line) cells [20].

## Conclusions

Kamishoyosan or Shakuyakukanzoto promotes neurite outgrowth with NGF via increasing the proportion of phosphorylated Erk1/2 and phosphorylated Akt in PC12 cells. We conclude that Kamishoyosan or Shakuyakukanzoto could likely be used safely and effectively in conjunction with paclitaxel chemotherapy, to rescue paclitaxel-induced neuropathy and ensure that the full round and dose intensity of chemotherapy is delivered to patients.

## Abbreviations

DMEM: Dulbecco’s modified Eagle medium
DTNB: 5,5′-dithiobis(2-nitrobenzoic acid)
Erk: Extracellular signal-regulated kinase
FBS: Fetal bovine serum
GAP-43: Growth-associated protein-43
HS: Horse serum
LY294002: 2-(4-Morpholinyl)-8-phenyl-1(4H)-benzopyran-4-one hydrochloride
NF-L: Light neurofilament protein
NGF: Nerve growth factor
PC12 cells: Rat adrenal pheochromocytoma cells
TJ107: Goshajinkigan
TJ24: Kamishoyosan
TJ68: Shakuyakukanzoto
U0126: 1,4-diamino-2,3-dicyano-1,4-bis[2-aminophenylthio] butadiene
